# SOX2 expression is associated with a cancer stem cell state and down-regulation of CDX2 in colorectal cancer

**DOI:** 10.1186/s12885-016-2509-5

**Published:** 2016-07-13

**Authors:** Ida V. Lundberg, Sofia Edin, Vincy Eklöf, Åke Öberg, Richard Palmqvist, Maria L. Wikberg

**Affiliations:** Department of Medical Biosciences, Pathology, Umeå University, Building 6M, SE-90185 Umeå, Sweden; Department of Surgical and Perioperative Sciences, Surgery, Umeå University, Umeå, Sweden

**Keywords:** SOX2, CDX2, Colorectal cancer, Prognosis, Cancer stem cell

## Abstract

**Background:**

To improve current treatment strategies for patients with aggressive colorectal cancer (CRC), the molecular understanding of subgroups of CRC with poor prognosis is of vast importance. SOX2 positive tumors have been associated with a poor patient outcome, but the functional role of SOX2 in CRC patient prognosis is still unclear.

**Methods:**

An in vitro cell culture model expressing SOX2 was used to investigate the functional role of SOX2 in CRC. In vitro findings were verified using RNA from fresh frozen tumor tissue or immunohistochemistry on formalin fixed paraffin embedded (FFPE) tumor tissue from a cohort of 445 CRC patients.

**Results:**

Using our in vitro model, we found that SOX2 expressing cells displayed several characteristics of cancer stem cells; such as a decreased proliferative rate, a spheroid growth pattern, and increased expression of stem cell markers CD24 and CD44. Cells expressing SOX2 also showed down-regulated expression of the intestinal epithelial marker CDX2. We next evaluated CDX2 expression in our patient cohort. CDX2 down-regulation was more often found in right sided tumors of high grade and high stage. Furthermore, a decreased expression of CDX2 was closely linked to MSI, CIMP-high as well as *BRAF* mutated tumors. A decreased expression of CDX2 was also, in a stepwise manner, strongly correlated to a poor patient prognosis. When looking at SOX2 expression in relation to CDX2, we found that SOX2 expressing tumors more often displayed a down-regulated expression of CDX2. In addition, SOX2 expressing tumors with a down-regulated CDX2 expression had a worse patient prognosis compared to those with retained CDX2 expression.

**Conclusions:**

Our results indicate that SOX2 expression induces a cellular stem cell state in human CRC with a decreased expression of CDX2. Furthermore, a down-regulated expression of CDX2 results in a poor patient prognosis in CRC and at least part of the prognostic importance of SOX2 is mediated through CDX2 down-regulation.

**Electronic supplementary material:**

The online version of this article (doi:10.1186/s12885-016-2509-5) contains supplementary material, which is available to authorized users.

## Background

Colorectal cancer (CRC) is a common malignancy worldwide and the second leading cause of cancer deaths in the western world [1]. CRC is often detected at late stages contributing to the high mortality rate seen in this disease. Today, most patients receive a similar stage specific treatment strategy, however not all benefit from it. In future treatment of CRC patients, personalized therapy will be of vast importance, but this will also place higher demands on the molecular subclassification of CRC.

The SOX2 gene encodes for a transcription factor and is a member of the SRY-related HMG-box (SOX) gene family. It is known that SOX2 plays essential roles in cell fate determination, thereby regulating developmental processes [2]. In recent years, aberrant expression of SOX2 has been reported in CRC as well as several other types of cancers [3–6]. According to our previous study, SOX2 expression was found to be correlated to high tumor grade, mutated *BRAF* and a poor patient prognosis [7]. We further found that the expression of SOX2 was partly regulated by BRAF [7]. Expression of SOX2 has also been associated with distant metastases in right-sided colon cancer [8], suggesting that SOX2 expressing tumors represent a subgroup with poor patient outcome. In CRC, SOX2 has previously been suggested to regulate epithelial-mesenchymal transition (EMT) and increased tumor migration and invasion [9]. However, the functional role of SOX2 in CRC patient prognosis is still unclear.

Recent research has revealed that a small subgroup of tumor cells possesses characteristics associated with stem cells and have therefore been called cancer stem cells (CSCs). CSCs have the ability of self-renewal and multi-lineage differentiation, features that can cause both tumor growth and emergence of new tumors [10–12]. SOX2 expression has been associated with a stem cell state in human ovarian, cervical, pancreatic, head and neck squamous cell, and breast carcinoma [3, 13–16], but so far this has not been shown in CRC. SOX2 expression has been associated with tumors of high grade (poorly differentiated) in different cancers [7, 17–20]. The transcription factor, Caudal type homeobox 2 (CDX2), is a major regulator of the expression of intestine-specific genes involved in cell differentiation [21, 22]. CDX2 is expressed at high levels in the normal colorectal epithelium, but loss or decrease of expression is seen in a subset of CRCs [23, 24]. Previous studies have also reported that loss of CDX2 is associated with poor patient prognosis in CRC [25–27].

In this study we investigated the functional role of SOX2 in CRC using an in vitro cell culture model. We found no evidence that SOX2 was involved in regulation of EMT or cellular migration. However, SOX2 positive cells were found to display several characteristics of cancer stem cells, as well as a decreased expression of the intestinal epithelial marker CDX2. In a cohort of CRC patients, we further demonstrate that SOX2 expression is significantly associated with down-regulated expression of CDX2 and at least part of the prognostic importance of SOX2 is mediated through CDX2 down-regulation. In conclusion, we suggest that CDX2 down-regulation is partly regulated by SOX2 and contributes to a poor prognosis in this patient group.

## Methods

### Cell culture and cell lines

In this study, the human colon cancer cell lines Caco2, SW480 and SW620 (American Type Culture Collection, Manassas, VA, USA) were grown in Dulbecco’s modified Eagle’s medium (DMEM) with glutamax supplemented with 10 % fetal bovine serum (FBS) (Gibco, Life Technologies, Stockholm, Sweden) and maintained at 37 °C and 5 % CO_2_. The stable transfectant expressing increased levels of SOX2 has been previously described [7].

### Migration assay

Cell migration was analyzed using transwell cell culture inserts with a pore size of 8 μm (BD Biosciences, Stockholm, Sweden) in 24-well plates. Caco2 or Caco2-SOX2 were seeded at a density of 1x10^5^ per insert in cell culture medium with 10 % FBS for 2–3 h. Media was subsequently exchanged to serum-free DMEM and cells were allowed to migrate towards either culture media supplemented with 10 % FBS or serum-free medium for 20 h at 37 °C and 5 % CO_2_. Cells remaining on the inside of the insert were removed with cotton swabs and the cells that migrated through the membrane were fixed and stained with Coomassie blue (Bio-Rad Laboratories, Solna, Sweden). For quantification, three fields were chosen randomly and migrating cells were counted at x10 magnification using a light microscope. The experiment was repeated three times.

### Proliferation assay

Cell proliferation was assessed by the XTT assay (Roche Diagnostics, Bromma, Sweden) according to the manufacturer’s instructions. In brief, Caco2 or Caco2-SOX2 cells were cultured in a 96-well plate at a density of 5x10^3^ per well in cell culture media supplemented with 10 % FBS for 72 h at 37 °C and 5 % CO_2_. The cells were then incubated with XTT labeling for 4 h at 37 °C before the absorbance was measured with an ELISA reader at a wavelength of 490 nm. A reference wavelength at 650 nm was also measured. Quadruplicates of each sample were analyzed and the experiment was repeated three times.

### Real time PCR

The NucleoSpin RNA kit (Macherey-Nagel, Duren, Germany) was used for isolation of total RNA from cultured cells, and cDNA was synthesized with the SuperScript II Reverse Transcriptase (Invitrogen, Life Technologies, Stockholm, Sweden). Fresh frozen human tumor tissue was homogenized using the gentleMACS Dissociator (Miltenyi Biotec, Bergisch Gladbach, Germany) before total RNA was isolated with the High Pure RNA Paraffin Kit (Roche Diagnostics, Stockholm, Sweden) and then converted into cDNA using the SuperScript VILO cDNA Synthesis Kit (Invitrogen, Life Technologies, Stockholm, Sweden). All steps were performed according to manufacturer’s protocols.

In the present study, primers for GAPDH, RPL13A, SOX2, MMP3, MMP11, E-cadherin, Snail and Fibronectin were from DNA Technology A/S (Aarhus, Denmark) and their sequences are listed in Additional file 1. For the remaining genes, Quantitect Primer Assays (Qiagen, Sollentuna, Sweden) were used. Quantitative RT-PCR reactions were performed on an ABI 7900HT instrument (Applied Biosystems, Life Technologies, Stockholm, Sweden) with the following thermal cycling conditions used: 50 °C for 2 min and then an initial denaturation at 95 °C for 10 min, followed by 40 cycles of 95 °C for 15 s and 60 °C for 60 s. Gene expression was normalized to GAPDH for cultured cells or RPL13A for fresh frozen tumor specimens. Standard deviations were calculated for the mean of triplicate reactions.

### Clinical samples

CRC specimens included in this study were from the Colorectal Cancer in Umeå Study (CRUMS) [28]. Tumor tissue samples were collected from patients with primary CRC that underwent tumor-resective surgery between 1995 and 2003 at Umeå University Hospital, Sweden. Formalin-fixed paraffin-embedded (FFPE) tissue was sampled from all patients and fresh frozen tumor tissue was collected from a subgroup of the patients. One pathologist did all histopathological classifications by reviewing routinely stained tumor sections, as previously described [28]. Clinical data were obtained from the patient records, and survival data was collected during autumn 2012. 445 cases were included in this study, but due to unavailable or insufficient tumor sample or negative staining in adjacent normal colon epithelium (*n* = 14), 431 of the tumors could be successfully analyzed for CDX2 expression.

Analyses of microsatellite instability (MSI) screening status, CpG island methylator phenotype (CIMP) status and mutational status of *BRAF* and *KRAS* have previously been described [29, 30]. In brief, MSI screening status was determined in FFPE tissue samples by immunohistochemical analyses of the expression of four mismatch repair proteins (MLH1, MSH2, MSH6 and PMS2). Tumors lacking nuclear staining for at least one of the four proteins were considered to have a positive MSI screening status, compared to those with a negative MSI screening status, referred to as microsatellite stable (MSS). CIMP status was determined by the MethyLight method with previously described primer and probe sequences. An eight gene panel (*CDKN2A, MLH1, CACNA1G, NEUROG1, RUNX3, SOCS1, IGF2,* and *CRABP1*) was used for evaluation of the hypermethylation status: CIMP-negative tumors (no promoter hypermethylation), CIMP-low tumors (one to five genes methylated) or CIMP-high tumors (six to eight genes methylated). *BRAF*^*V600E*^ mutation was detected by the Taqman allelic discrimination assay [31] (reagents from Applied Biosystems, Life Technologies, Stockholm, Sweden). *KRAS* mutational status was determined by sequencing using Big Dye v. 3.1 (Applied Biosystems, Life Technologies, Stockholm, Sweden). The expression of SOX2 has previously been evaluated in this patient cohort [7], where nuclear staining was assessed as either negative or positive.

### Immunohistochemistry

FFPE CRC specimens were cut at 4-μm and then dried, deparaffinized and rehydrated. CDX2 mouse monoclonal antibody (clone CDX2-88, Biogenex, Fremont, CA, USA) was used at a dilution of 1:50 and visualized by the iVIEW DAB Detection kit on an Ventana Benchmark Ultra staining machine (Ventana Medical Systems, Tucson, AZ, USA), with the CC1 standard pretreatment. Normal colon mucosa was used as positive control. The slides were counterstained with hematoxylin. Immunohistochemical staining of CDX2 was evaluated under light microscopy by one observer two times under supervision of an experienced pathologist. In cases with discrepant scoring, a third final evaluation was made. Nuclear CDX2 staining was scored as: <5 % positive tumor cells, 5-50 % positive tumor cells or >50 % positive tumor cells. Normal colon mucosa, if included in the sample, was used as an internal positive control.

### Statistical analyses

IBM SPSS Statistics software version 21 (SPSS Inc., Chicago, Illinois, USA) was used for statistical analyses. The nonparametric Mann–Whitney *U*-test was performed in order to compare the differences in gene expression levels between two groups. Cross-tabulations for associations between CDX2 expression and different clinicopathological and molecular variables were analyzed with *χ*^2^ tests. To estimate cancer-specific survival, Kaplan-Meier survival analysis was used, and the log-rank test was used for comparisons between groups. Cancer-specific survival was defined as death with known disseminated or recurrent disease. Patients lacking survival data or patients who died with postoperative complications within one month after surgery (*n* = 34) were excluded from the survival analyses. For multivariable analyses, Cox proportional hazard models were used. *P* < 0.05 was considered statistically significant for all analyses.

## Results

To gain mechanistic insights to the prognostic importance of SOX2 in CRC, we created a stable transfectant of the CRC cell line Caco2 expressing increased levels of SOX2 (Caco2-SOX2) as previously described [7]. The Caco2 cell line was chosen to represent one of the largest subgroups of sporadic CRCs; CIMP negative, MSS and wild-type in KRAS and BRAF [32].

### SOX2 is not a major regulator of EMT and cellular migration in CRC cells

**Fig. 1:**
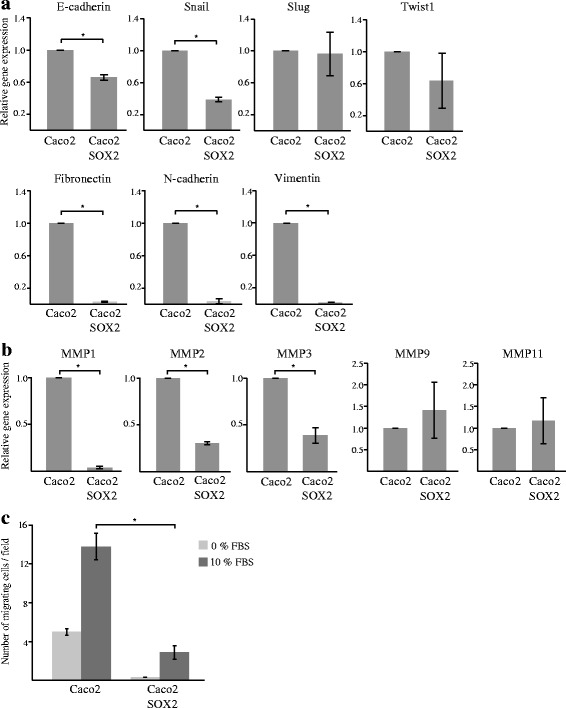
Evaluation of EMT and cellular migration in response to SOX2 expression. Caco2 cells and Caco2 cells stably transfected with SOX2 (Caco2-SOX2) was analyzed by RT-PCR for the expression of **a** EMT related genes, or **b** MMPs. Shown is relative gene expression from three or more independent experiments ± SD with Caco2 levels set as 1. **c** Cellular migration was evaluated using Boyden chamber experiments. Shown is mean number of migrating cells ± SD from three independent experiments. Significant differences are indicated by * (*P* < 0.05)

In CRC, SOX2 has previously been linked to EMT and increased migration and invasion [9]. EMT is linked to changes in expression of several transcription factors and cellular adhesion molecules. To investigate these events in our in vitro model, we compared the expression of EMT related factors in Caco2 and Caco2-SOX2 cells. Even though the epithelial marker E-cadherin (CDH1) was found to be significantly decreased by SOX2, the expression of the transcription factors Snail (SNAI1), Slug (SNAI2) and Twist1, controlling E-cadherin expression were unaltered or decreased (Fig. 1a). Furthermore, expression of Fibronectin, N-cadherin and Vimentin, associated with a mesenchymal phenotype, were not increased but instead severely decreased in SOX2 expressing cells (Fig. 1a). We also investigated the expression of several matrix metalloproteinases (MMPs) to examine possible effects on the extracellular matrix, caused by SOX2 expression. Most of the investigated MMPs were found to be down-regulated in Caco2-SOX2 cells (Fig. 1b). We further compared the migratory ability of Caco2 and Caco2-SOX2 cells using Boyden transwell migration experiments. Cellular migration was found to be significantly decreased in Caco2 cells expressing SOX2 compared to Caco2 wild type cells (Fig. 1c). Together, these results incline that SOX2 is not a major regulator of EMT or cellular migration and invasion in our in vitro model.

### SOX2 induces a stem cell state in CRC cells

**Fig. 2:**
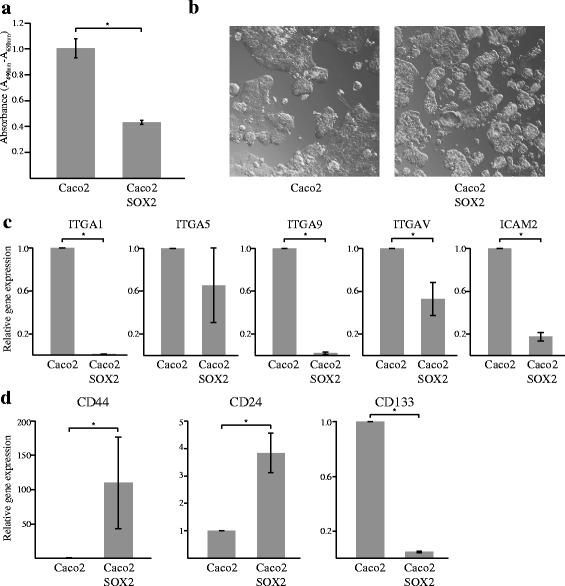
SOX2 induces a CSC state in CRC cells. Factors associated with a CSC state was evaluated in Caco2 cells and Caco2 cells stably transfected with SOX2 (Caco2-SOX2). **a** Proliferation of cells as measured by XTT cell proliferation assay. **b** Morphological evaluation of cells. **c** Expression of cellular adhesion molecules as evaluated by RT-PCR. Shown is relative expression with Caco2 cells set as 1. **d** Expression of cancer stem cell markers as evaluated by RT-PCR. Shown is relative expression with Caco2 cells set as 1. Gene expression analyses were reproduced three times and mean values ± SD is shown. Significant differences are indicated by * (*P* < 0.05)

Further investigations revealed that Caco2 cells expressing high levels of SOX2 had a lower proliferative rate (Fig. 2a), were less adherent and displayed a spheroid growth pattern compared to Caco2 wild type cells that were more confluent (Fig. 2b). Accordingly, Caco2-SOX2 cells showed decreased expression of several important adhesion molecules (Fig. 2c). Decreased proliferation and adhesion are events indicative of a cancer stem cell state. We further analyzed cancer stem cell markers, CD44, CD24 and CD133, associated with aggressive cancer types and poor prognosis in CRC [33] (Fig. 2d). Expression of CD44 and CD24 was found to be significantly increased in Caco2 cells expressing SOX2. The levels of CD133 were instead found to be decreased. Together, the phenotype seen in Caco2-SOX2 cells suggests that SOX2 might induce a cancer stem cell state in CRC leading to increased aggressiveness and poorer patient prognosis.

### SOX2 is inversely associated with expression of the intestinal epithelial cell marker CDX2

**Fig. 3:**
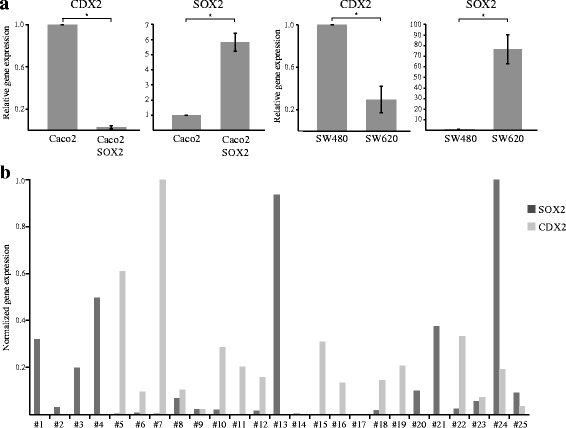
SOX2 is associated with down-regulated expression of CDX2. **a** Caco2, Caco2-SOX2, SW480 and SW620 cells were analyzed by RT-PCR for the expression of CDX2 and SOX2. Shown is relative gene expression from three independent experiments ± SD with Caco2 or SW480 levels set as 1. Significant differences are indicated by * (*P* < 0.05). **b** RNA from fresh frozen tumor tissue from 25 CRC patients was analyzed by RT-PCR for the expression of SOX2 and CDX2. Shown is normalized gene expression

We previously found that SOX2 positive CRC tumors more often are poorly differentiated [7]. A poor cell differentiation is also found in tumors that loose the expression of CDX2, an intestine-specific transcription factor essential for intestinal homeostasis and for the maintenance of an intestinal epithelial phenotype [34]. Furthermore, loss of CDX2 has been linked to more aggressive tumors and a poor outcome in CRC [25–27]. We compared the expression of SOX2 and CDX2 in Caco2 and Caco2-SOX2 cells. We found that Caco2-SOX2 cells showed significantly decreased levels of CDX2, compared to control Caco2 cells (Fig. 3a). Furthermore, we compared SW480 and SW620 CRC cell lines, derived from a primary and metastatic tumor, respectively, resected from a single patient. A high expression of SOX2 was found in the metastatic cell line, and correlated to a significantly decreased expression of CDX2 (Fig. 3a). We further analyzed fresh frozen tumor tissue from 25 CRC patients by RT-PCR for the expression of SOX2 and CDX2. Generally, in tumors with a high expression of SOX2 the levels of CDX2 were either low or absent (Fig. 3b). Likewise, in tumors with a high expression of CDX2, the levels of SOX2 were low or absent (Fig. 3b). These findings suggest that SOX2 expression is associated with a down-regulated expression of CDX2.

### SOX2 is associated with a down-regulated expression of CDX2 in CRC patients

**Fig. 4:**
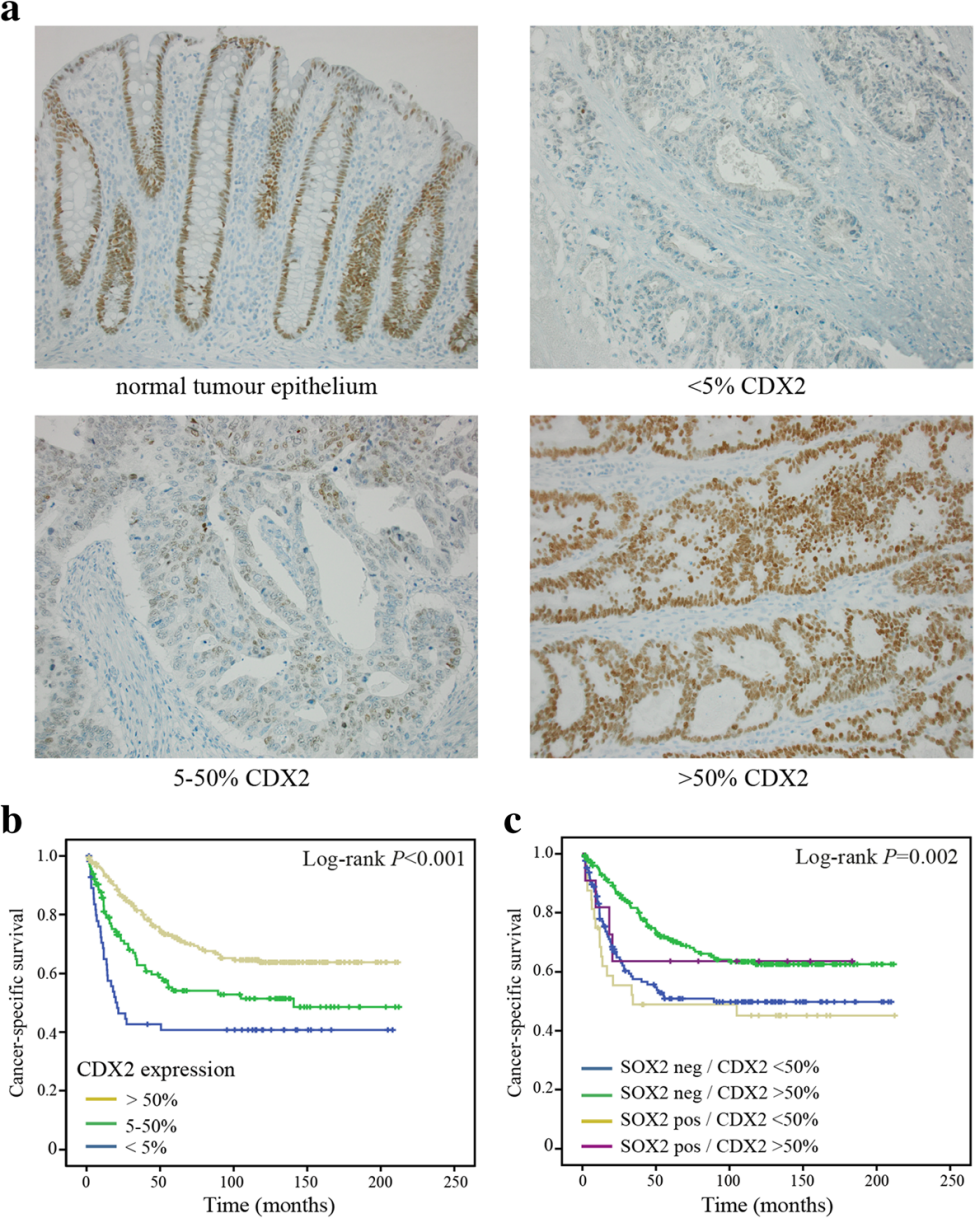
Evaluation of CDX2 expression in CRC. **a** Representative images of immunohistological stainings of CDX2 in human CRC tissue specimens; normal colon epithelium and CRC with <5 % expression, 5–50 % expression and >50 % expression of CDX2. **b** Kaplan-Meier survival analyses of CDX2 expression in CRC. **c** Kaplan-Meier survival analyses in subgroups of CRC defined as SOX2 positive or negative, and CDX2 < 50 % expression and >50 % expression. Log-rank tests were used to calculate *P*-values

The expression of CDX2 was evaluated by immunohistochemistry in a large cohort of 445 CRC patients. Nuclear CDX2 expression was scored in tumor tissue as; less than 5 % positive cells, 5–50 % positive cells or, more than 50 % positive cells. Representative images of the immunohistochemical stainings of CDX2 can be found in Fig. 4a. In total, 43.4 % of patients showed less than 50 % CDX2 positive tumor nuclei, and of those 14.4 % showed a close to complete lack of CDX2 expression (<5 % positive cells) (Table 1). A down-regulated expression of CDX2 was more often found in right sided tumors (*P* < 0.001) and tumors of higher stage (*P* < 0.001) (Table 1). Furthermore, loss of CDX2 expression was significantly associated with poorly differentiated tumors (*P* < 0.001) (Table 1). In survival analyses, a down-regulated expression of CDX2 correlated, in a stepwise manner, to a poor patient survival (*Log-rank P* < 0.001) (Fig. 4b). The prognostic importance of CDX2 down-regulation stayed significant in multivariable Cox regression analyses adjusting for stage, age, sex, localization and grade (for patients with 5–50 % CDX2 (HR = 1.54, 95 % CI 1.04–2.28, *P* = 0.031) and for patients with <5 % CDX2 (HR = 2.45, 95 % CI 1.50–4.01, *P* < 0.001)).

**Table 1:** CDX2 expression in relation to clinicopathological characteristics in CRC

	CDX2 expression			
	<5 %	5–50 %	>50 %	*P* value^a^
Frequencies, n (%)	62 (14.4)	125 (29.0)	244 (56.6)	
Sex, n (%)				0.667
Male	33 (14.3)	63 (27.3)	135 (58.4)	
Female	29 (14.5)	62 (31.0)	109 (54.5)	
Age, n (%)				0.275
≤59 years	16 (21.1)	18 (23.7)	42 (55.3)	
60–69 years	16 (14.7)	29 (26.6)	64 (58.7)	
70–79 years	20 (12.9)	54 (34.8)	81 (52.3)	
≥80 years	10 (11.0)	24 (26.4)	57 (62.6)	
TNM stage, n (%)^b^				<0.001
I	2 (3.0)	21 (31.8)	43 (65.2)	
II	19 (11.2)	36 (21.3)	114 (67.5)	
III	17 (19.3)	32 (36.4)	39 (44.3)	
IV	24 (24.2)	33 (33.3)	42 (42.4)	
Localization, n (%)^b^				<0.001
Right colon	41 (29.5)	27 (19.4)	71 (51.1)	
Left colon	9 (7.1)	39 (30.7)	79 (62.2)	
Rectum	12 (7.5)	59 (36.6)	90 (55.9)	
Grade, n (%)^b^				<0.001
Highly to moderately differentiated	13 (6.3)	45 (22.0)	147 (71.7)	
Moderately to poorly differentiated	49 (22.5)	80 (36.7)	89 (40.8)	

^a^χ^2^ test

^b^The following numbers of missing cases were present: TNM stage, 9; localization, 4; grade, 8

When looking at molecular subgroups of CRC, a down-regulated expression of CDX2 was closely linked to CIMP-high (*P* < 0.001), MSI (*P* < 0.001) and *BRAF* mutated tumors (*P* < 0.001) (Table 2). Furthermore, a down-regulated expression of CDX2 was more often found in SOX2 positive tumors (*P* < 0.001) (Table 2). Of the SOX2 positive tumors, 73.9 % had less than 50 % CDX2 positive tumor cells, and of these 32.6 % had less than 5 % positive cells (Table 2).

**Table 2:** CDX2 expression in relation to molecular characteristics in CRC

	CDX2 expression			
	<5 %	5–50 %	>50 %	*P* value^a^
Frequencies, n (%)	62 (14.4)	125 (29.0)	244 (56.6)	
MSI screening status, n (%)^b^				<0.001
MSI	27 (41.5)	14 (21.5)	24 (36.9)	
MSS	33 (9.4)	108 (30.8)	210 (59.8)	
CIMP status, n (%)^b^				<0.001
CIMP-negative^c^	8 (3.8)	66 (31.1)	138 (65.1)	
CIMP-low^c^	25 (15.6)	46 (28.8)	89 (55.6)	
CIMP-high^c^	29 (52.7)	10 (18.2)	16 (29.1)	
*BRAF* ^*V600E*^, n (%)^b^				<0.001
wild type	27 (7.4)	110 (30.3)	226 (62.3)	
mutated	35 (58.3)	11 (18.3)	14 (23.3)	
*KRAS* (codon 12, 13), n (%)^b^				0.027
wild type	55 (16.2)	88 (26.0)	196 (57.8)	
mutated	7 (8.2)	33 (38.8)	45 (52.9)	
SOX2 expression, n (%)^b^				<0.001
SOX2 negative	44 (12.3)	100 (27.9)	215 (59.9)	
SOX2 positive	15 (32.6)	19 (41.3)	12 (26.1)	

Abbreviations: *MSI* microsatellite instability, *MSS*, microsatellite stable, *CIMP* CpG island methylator phenotype (according to an eight-gene CIMP panel)

^a^χ^2^ test

^b^The following numbers of missing cases were present: MSI screening status, 15; CIMP status, 4; BRAF V600E, 8; KRAS, 7; SOX2 expression, 26

^c^CIMP negative, no promoter hypermethylation; CIMP low, one to five genes methylated; CIMP high, six to eight genes methylated

SOX2 positive tumors with a down-regulated expression of CDX2 (<50 % positive cells) had a worse prognosis than SOX2 positive tumors with retained CDX2 expression (>50 % positive cells) (Fig. 4c), suggesting that a part of the negative role of SOX2 on prognosis might be through down-regulation of CDX2. 40.2 % of the SOX2 negative tumors also showed a down-regulated expression of CDX2 (less than 50 % positive cells) (Table 2). In SOX2 negative tumors, CDX2 down-regulation also resulted in a poorer patient prognosis (Fig. 4c), suggesting that CDX2 expression in itself is a strong prognostic factor and likely can be regulated also independently of SOX2.

## Discussion

Expression of SOX2 is associated with a poor patient prognosis in CRC, but so far the molecular mechanisms have not been fully elucidated. Here we have studied the functional role of SOX2 in CRC. Using an in vitro cell culture model, we could show that expression of SOX2 was associated with a cellular stem cell state and decreased expression of the intestinal epithelial marker CDX2. The correlation of SOX2 expression and a decreased expression of CDX2 could further be verified in our patient cohort. We also found that decreased expression of CDX2 correlated to a poor patient survival, and that SOX2 expressing tumors with a down-regulated CDX2 expression had a worse patient prognosis compared to those with a retained CDX2 expression, suggesting that a part of the negative role of SOX2 on prognosis might be through down-regulation of CDX2.

In a previous report by Han et al., a role for SOX2 in EMT and increased migration and invasion in CRC was presented [9]. In our in vitro model expressing SOX2, we found that SOX2 did not increase the migratory effect of tumor cells and was not a major regulator of EMT. Instead, our cells expressing SOX2 showed a lower proliferative rate, were less adherent and displayed a more spheroid growth pattern compared to wild type cells. These are all characteristics of CSCs. CSCs possess stem cell-like features, like the ability of self-renewal and multi-lineage differentiation and they have been proposed to retain their tumorigenic capacity and to be the cells responsible for initiation, maintenance and spreading of the tumor [35, 36]. Sphere-forming CSCs have been shown to be more aggressive (metastatic) in vivo than adherent cells [37]. Stem cells divide more slowly than differentiated cells, and the quiescent slow-cycling phenotype seen in CSCs probably plays a role in tumor recurrence as well as resistance to treatment [38, 39]. A possible SOX2 induced CSC state in our in vitro model could thereby be one explanation to the decreased survival seen in patients with SOX2 positive tumors. In line with this hypothesis, cancer cells expressing SOX2 showed an increased expression of the stem cell markers CD24 and CD44. The expression of the stem cell marker CD133 was instead decreased. CD24 has previously been shown to be regulated by SOX2 [5]. CD44 has been identified as a potential CSC marker in CRC [40] and has also been shown to be a more selective colon CSC marker than CD133 since decreased expression of CD44, but not CD133, has been shown to reduce both clonal formation and tumor formation [41]. Other studies have also indicated that CD133 might not be a good CSC marker in CRC, since knocking-down the gene expression of CD133 does not induce cellular differentiation in CRC [42], and both CD133 positive and CD133 negative CRC subpopulations are capable of tumor initiation [43]. CD44 is also the main receptor of the ECM component hyaluronan [44], and it has been shown that expression of CD44 on tumor cells correlate with cancer cell adhesion to endothelial cells and also with metastasis [45]. In a previous study, a cluster of stem-like factors, including SOX2 and CD44, identified patients with a worse prognosis [46]. We plan to further investigate the role of SOX2 in cancer stem cell differentiation and tumor progression. In few previous studies, the suggested role of SOX2 in cancer stem cell differentiation has, in difference to our study, at least partly been linked to EMT related factors [14, 15]. Further studies of the functional role of SOX2 in human cancers are required to clarify these differences.

In our previous study of SOX2 expression in CRC, we have shown that poorly differentiated tumors more often are SOX2 positive [7]. Poorly differentiated tumors have also been associated with decreased expression of the intestinal epithelial marker CDX2 (reviewed in [47]). Therefore, we were interested in studying the expression of CDX2 in correlation to expression of SOX2. Two different cell lines expressing SOX2 showed a decreased expression of CDX2 compared to the wild type cell lines. RT-PCR analyses of fresh frozen tumor tissue verified that tumors with a high expression of SOX2 had low or absent expression of CDX2. When analyzing the expression of CDX2 by immunohistochemistry in our patient cohort, we found that SOX2 positive tumors were highly associated with a decreased expression of CDX2; 73.9 % of the SOX2 positive tumors had less than 50 % CDX2 positive tumor cells, and of these 32.6 % had less than 5 % positive cells. Together these results suggest that expression of SOX2 is correlated to a down-regulated expression of CDX2.

A down-regulated expression of CDX2 in CRC has previously been linked to subgroups of tumors defined as CIMP-high, MSI, and *BRAF* mutated although results are still inconclusive (reviewed in [47]). In our cohort, a down-regulated expression of CDX2 was closely linked to CIMP-high (*P* < 0.001), MSI (*P* < 0.001) and *BRAF* mutated tumors (*P* < 0.001) (Table 2), strengthening previously published findings. This finding is also in line with our previous results that SOX2 is partly regulated by BRAF [7]. Down-regulated expression of CDX2 was also correlated to a poor patient prognosis in our cohort, similar to other reports in CRC [25–27]. In our study, the prognostic importance of CDX2 remained significant in multivariable analyses adjusted for stage and other confounders, suggesting that CDX2 is a powerful prognostic factor in CRC. When combining the expression of CDX2 and SOX2 we found that SOX2 positive tumors with a decreased expression of CDX2 had a worse patient prognosis compared to those with a retained CDX2 expression, indicating that at least a part of the negative role of SOX2 on prognosis might be through down-regulation of CDX2. This subgroup, showing SOX2 expression but a down-regulated expression of CDX2, has previously been shown to predict a worse patient outcome in gastric cancer [48]. Since decreased expression of CDX2 also could be seen in SOX2 negative tumors, we speculate that CDX2 can be down-regulated both by SOX2 dependent and SOX2 independent mechanisms. SOX2 expression is often seen only in a part of the tumor, and since SOX2 expression is analyzed in just one tissue section per tumor [7], the number of SOX2 positive tumors in our patient cohort may be underestimated and some of the SOX2 negative tumors with down-regulated expression of CDX2 might actually be SOX2 positive. However, likely there are also other events involved.

CDX2 has been suggested to be a target gene of the Hippo pathway in CRC [49]. The Hippo pathway normally plays critical roles in cell proliferation, growth and apoptosis, but when deregulated it is instead involved in initiation and progression of tumors [50, 51]. Studies have also suggested that SOX2 is involved in the deregulation of the Hippo pathway [52, 53], and therefore we speculate that the SOX2 mediated down-regulation of CDX2 might partly be through this pathway. Kuzmichev et al. have further shown that SOX21 can repress the expression of CDX2 in CRC, and that SOX21 is induced by SOX2 [54]. SOX21 might therefore be another possible pathway that SOX2 utilizes to regulate the expression CDX2. The mechanisms behind the regulation of CDX2 expression by SOX2 will be further investigated.

## Conclusions

Using an in vitro cell model, we found that SOX2 in CRC induces a CSC state with down-regulated expression of the intestinal epithelial transcription factor CDX2. In our patient cohort, the expression of SOX2 was highly and significantly associated with a down-regulated expression of CDX2. Furthermore, SOX2 expressing tumors with down-regulated expression of CDX2 had a particularly poor prognosis. We suggest that the poor prognosis seen in patients with SOX2 positive tumors is at least partly mediated through down-regulated expression of CDX2.

## Abbreviations

CDX2, caudal type homeobox 2; CIMP, CpG island methylator phenotype; CRC, colorectal cancer; CRUMS, Colorectal Cancer in Umeå Study; CSC, cancer stem cell; DMEM, Dulbecco’s modified Eagle’s medium; EMT, epithelial-mesenchymal transition; FBS, fetal bovine serum; FFPE, formalin-fixed paraffin-embedded; MMP, matrix metalloproteinase; MSI, microsatellite instability; MSS, microsatellite stable; SOX, SRY-related HMG-box

## Additional file

Additional file 1: Sequences of primers used for RT-PCR. (DOCX 17 kb)
