# Supplementary material for: SOX2 expression is associated with a cancer stem cell state and down-regulation of CDX2 in colorectal cancer
Source: BMC Cancer. 2016 Jul 13;16:471. doi: 10.1186/s12885-016-2509-5 (PMC4944515; doi:10.1186/s12885-016-2509-5)
Supplement: Additional file 1: — Sequences of primers used for RT-PCR. (DOCX 17 kb) [file 12885_2016_2509_MOESM1_ESM.docx]

| **Additional file 1**. Sequences of primers used for RT-PCR | |
| --- | --- |
| Name | Primer sequence |
| GAPDH | Forward: 5′-TGCACCACCAACTGCTTAGC-3′ |
|  | Reverse: 5′-GGCATGGACTGTGGTCATGAG-3′ |
| RPL13A | Forward: 5´-GTACGCTGTGAAGGCATCAA-3´ |
|  | Reverse: 5`-GTTGGTGTTCATCCGCTTG-3´ |
| SOX2 | Forward: 5′-AACCCCAAGATGCACAACTC-3′ |
|  | Reverse: 5′-CGGGGCCGGTATTTATAATC-3′ |
| MMP3* | Forward: 5′-TTCCTGGCATCCCGAAGTGG-3′ |
|  | Reverse: 5′-ACAGCCTGGAGAATGTGAGTGG-3´ |
| MMP11* | Forward: 5′-AGGGCCACATTTGGTTCTTC-3′ |
|  | Reverse: 5′-CAGCATGGACCGGGAACCT-3´ |
| E-cadherin | Forward: 5′-GTCGTCACCACAAATCCAG-3′ |
|  | Reverse: 5′-GTAGAATGTACTGCTGCTTGG-3´ |
| Snail | Forward: 5′-AAGCCCTCCGACCCCAATC -3′ |
|  | Reverse: 5′-GCTGCTGGAAGGTAAACTCTGG-3´ |
| Fibronectin | Forward: 5′-GTGTGACCCTCATGAGGCAAC -3′ |
|  | Reverse: 5′-CTGGCCTCCAAAGCATGTG-3´ |
| *MMP, matrix metalloproteinase | |
